# An Unusual Case of CD5-Positive Extranodal Marginal Zone Lymphoma of the Mucosa-Associated Lymphoid Tissue (MALT) Involving the Appendix

**DOI:** 10.7759/cureus.67883

**Published:** 2024-08-27

**Authors:** Neelayadakshi B, Sarah Grace Priyadarshini, Jayaganesh P, Vimal Chander R

**Affiliations:** 1 Pathology and Laboratory Medicine, Saveetha Medical College and Hospital, Saveetha Institute of Medical and Technical Sciences, Saveetha University, Chennai, IND; 2 Pathology, Saveetha Medical College and Hospital, Saveetha Institute of Medical and Technical Sciences, Saveetha University, Chennai, IND

**Keywords:** non-hodgkin lymphoma, thrombo-occlusive disease, extranodal malt lymphoma, right hemicolectomy, appendicular mass

## Abstract

Non-Hodgkin lymphoma (NHL) includes a diverse group of hematological malignancies. The common site for extranodal involvement of NHL is the gastrointestinal tract (GIT), with the stomach being the most prevalent site. The appendix is a very unusual site of involvement in NHL. This case report describes an uncommon instance of an appendicular mass in an elderly female who complained of vomiting for two weeks, as well as abdominal pain, and was radiologically suspected to have appendicular malignancy. A right hemicolectomy was done, and she was diagnosed with extranodal mucosa-associated lymphoid tissue (MALT) lymphoma involving the appendix. Postoperatively, she also developed thrombo-occlusive disease in her right lower limb, with right foot dry gangrene, for which thromboembolectomy was done. Later, a below-knee amputation was carried out. Lymphoma associated with thrombophilia is a rare presentation and not many cases have been reported in the literature. We present this case here on account of the rarity of lymphoma involving the appendix with associated thrombophilia.

## Introduction

Malignant lymphomas refer to a class of neoplasms that are derived from cells of the lymphoid tissue during any of their stages of development [1]. The two main categories of lymphomas that are commonly identified include non-Hodgkin lymphoma (NHL) and Hodgkin lymphoma [2]. Hodgkin and non-Hodgkin lymphomas commonly involve the head and neck region. However, extranodal illness - with or without lymph node involvement - is more prevalent in NHL patients [1]. 

NHL accounts for about 85% of all lymphomas. NHL includes a diverse group of hematological malignancies, having different histological subtypes [2]. Follicular lymphoma (approximately 20%) and diffuse large B-cell lymphoma (30%) are the most prevalent NHL subtypes seen in developed nations. The frequency of the remaining NHL subtypes accounts for less than 10%. NHL ranks sixth among the deaths occurring due to cancer in the United States, following colorectal, prostate, breast, lung, and bladder cancers [1]. Though the age-standardized incidence rates are modest (2.4 per 100,000) in comparison to rates in other regions of the world, India reports about 23,718 new NHL cases annually [3].

The gastrointestinal system (GIT) is the site where extranodal involvement in NHL occurs most frequently. The stomach is the most frequently affected part of the GIT, followed by the colon and small intestine [4]. However, the appendix is a rather uncommon location for NHL involvement [5], with an estimated incidence of 0.015% [6-8].

A 78-year-old female patient came to the hospital with an appendicular mass. After a right hemicolectomy, the specimen was sent for histological analysis and subsequent immunohistochemical staining (IHC). Features were conclusive of CD5-positive extranodal mucosa-associated lymphoid tissue (MALT) lymphoma involving the appendix and the adjacent caecal wall. One month later, she developed thrombo-occlusive disease in her right lower limb, for which thromboembolectomy was done. Following the surgery, the patient developed right foot dry gangrene, hence a below-knee amputation was carried out. However, she succumbed to the illness. This case is presented here because of the rarity of appendicular involvement by lymphoma and associated thrombophilia.

## Case presentation

An elderly female patient in her 70s came to the outpatient department with complaints of pain in the abdomen, vomiting, and blood-stained stools associated with mucus for about two weeks. A colonoscopy was done, which showed a smooth mass at the appendicular orifice and unhealthy mucosa in the caecum. CT abdomen with contrast was done, which showed a fairly defined, heterogeneously enhancing lesion in the caecum extending up to the tip of the appendix, suggestive of appendicular malignancy.

A right hemicolectomy with ileocolic anastomosis was done, and the specimen was sent to the pathology department for histopathological analysis.

**Figure 1:**
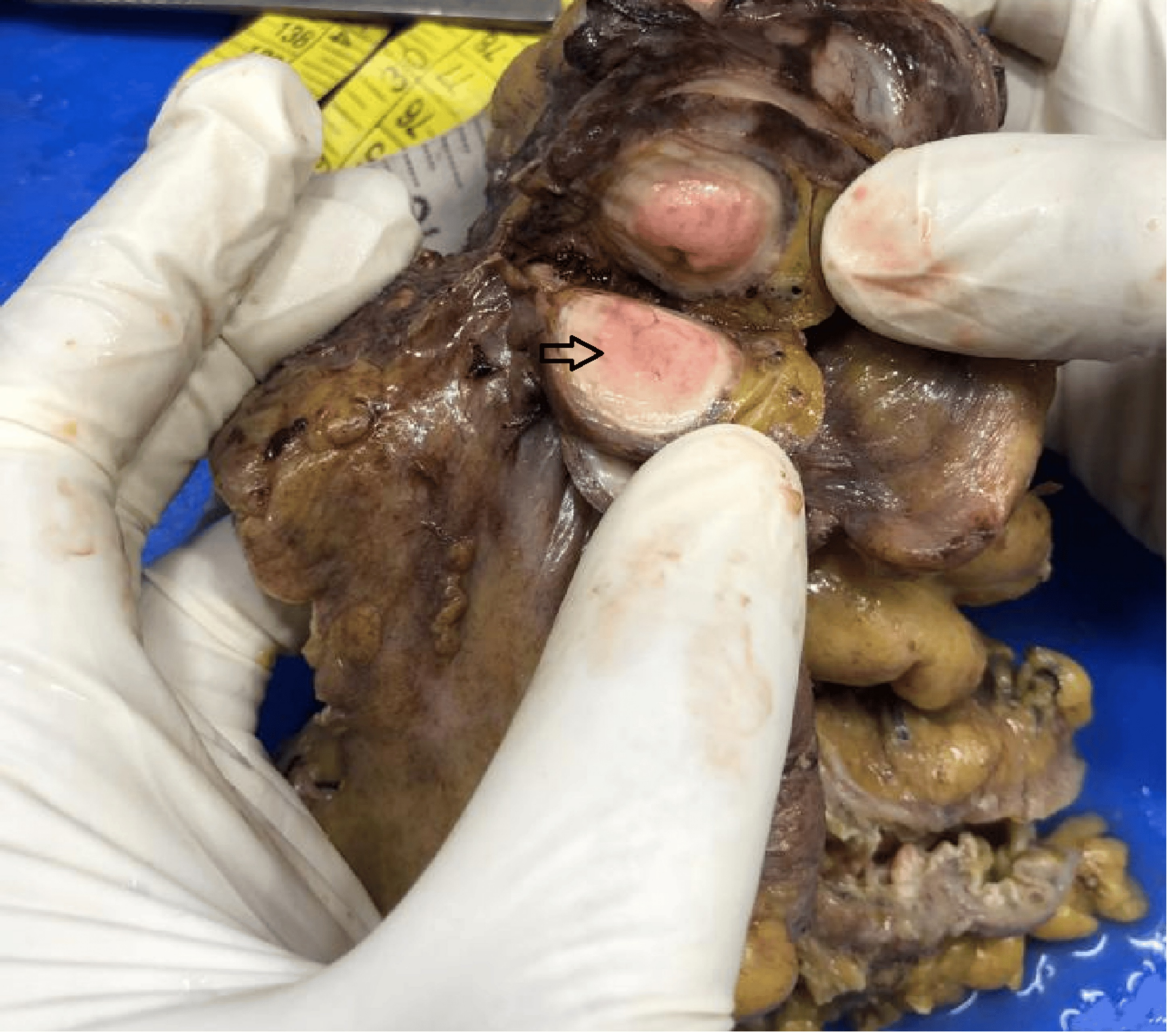
Gross cut section of the appendix with the tumor The grossly cut section of the appendix showed a grey-white homogenous mass, as indicated by the black arrow. The mass measured 7x5.5x4.5 cm in size. It was firm in consistency.

Grossly, the appendix showed a mass, measuring 7x5.5x4.5 cm in size, indenting the wall of the caecum. Two large lymph nodes were seen in the peri-appendicular fat, each measuring 1.2 cm in diameter (Figure 1).

**Figure 2:**
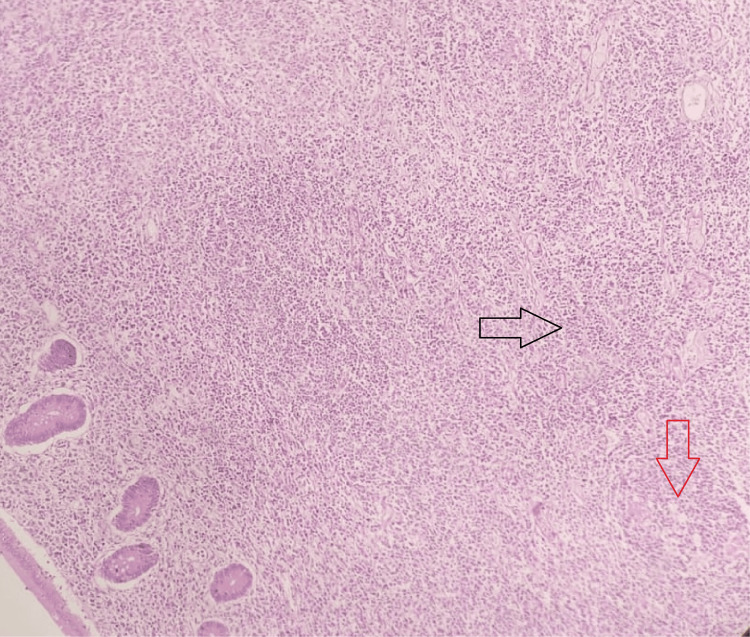
Microscopy - histopathological examination of tumor in the appendix (scanner view, H&E stain) This scanner view image shows appendicular mucosa with a lymphoid follicle and an expanded interfollicular region by sheets of small lymphoid cells. The red arrow denotes the follicle, and the black arrow denotes the expanded interfollicular region by sheets of small lymphoid cells.

**Figure 3:**
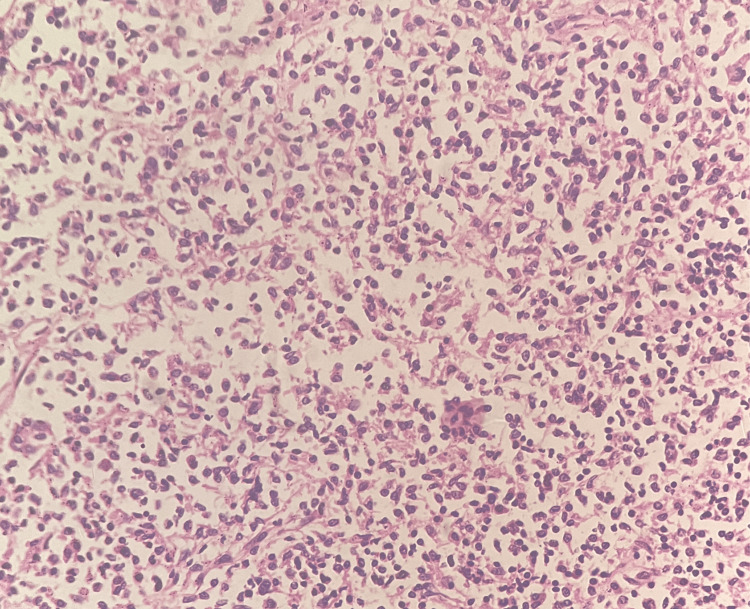
Microscopy - histopathological examination of tumor in the appendix (20x magnification, H&E stain) This section shows sheets of small lymphoid cells having an irregular nuclear membrane with a scant amount of cytoplasm.

Microscopy showed appendicular mucosa with ulceration overlying a few lymphoid follicles with germinal centers and an expanded interfollicular region by sheets of small lymphoid cells (Figure 2) having an irregular nuclear membrane with a scant amount of cytoplasm (Figure 3) and admixed with plenty of plasma cells infiltrating into the muscularis propria layer and peri-appendicular fat. The cells also infiltrated into the adjacent caecal wall. Sections from one of the lymph nodes showed partial architectural effacement by similar lymphoplasmacytic infiltrate.

**Figure 4:**
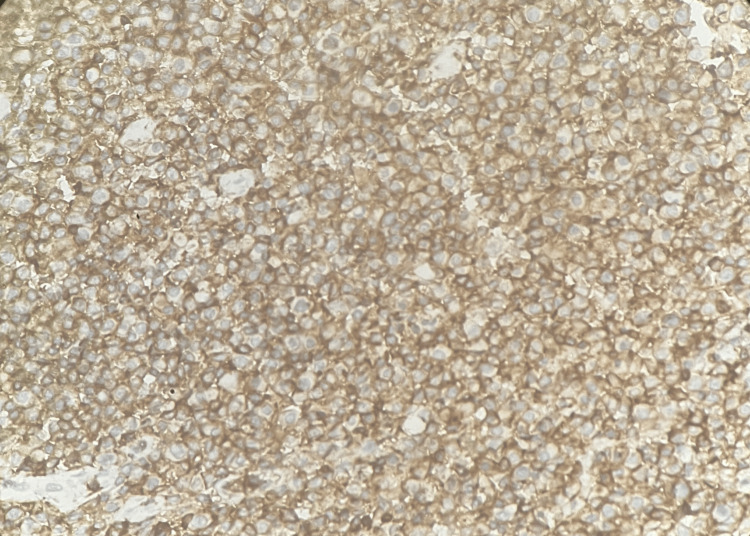
Tumor in the appendix - CD45 immunohistochemistry (20x magnification) CD45 showed strong membranous positivity in 100% of lymphoid cells.

Sections that were formalin-fixed and paraffin-embedded were subjected to IHC utilizing the streptoavidin-biotin (SAB) technique. CD45 showed strong membranous positivity in 100% of lymphoid cells (Figure 4).

**Figure 5:**
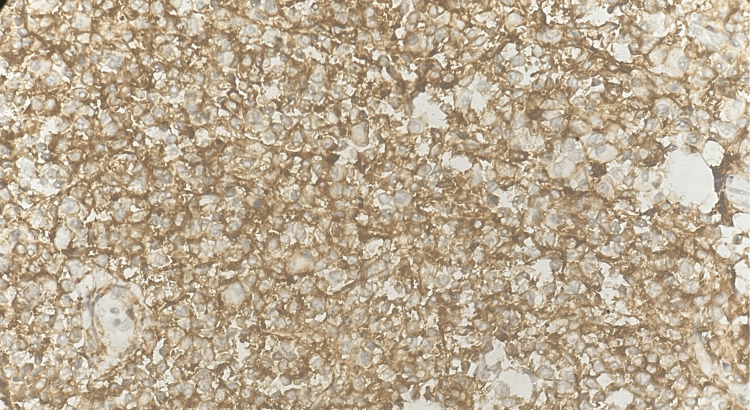
Tumor in the appendix - CD20 immunohistochemistry (10x magnification) CD20 showed strong membranous positivity in 90% of lymphoid cells.

CD20 showed strong membranous positivity in 90% of lymphoid cells (Figure 5).

**Figure 6:**
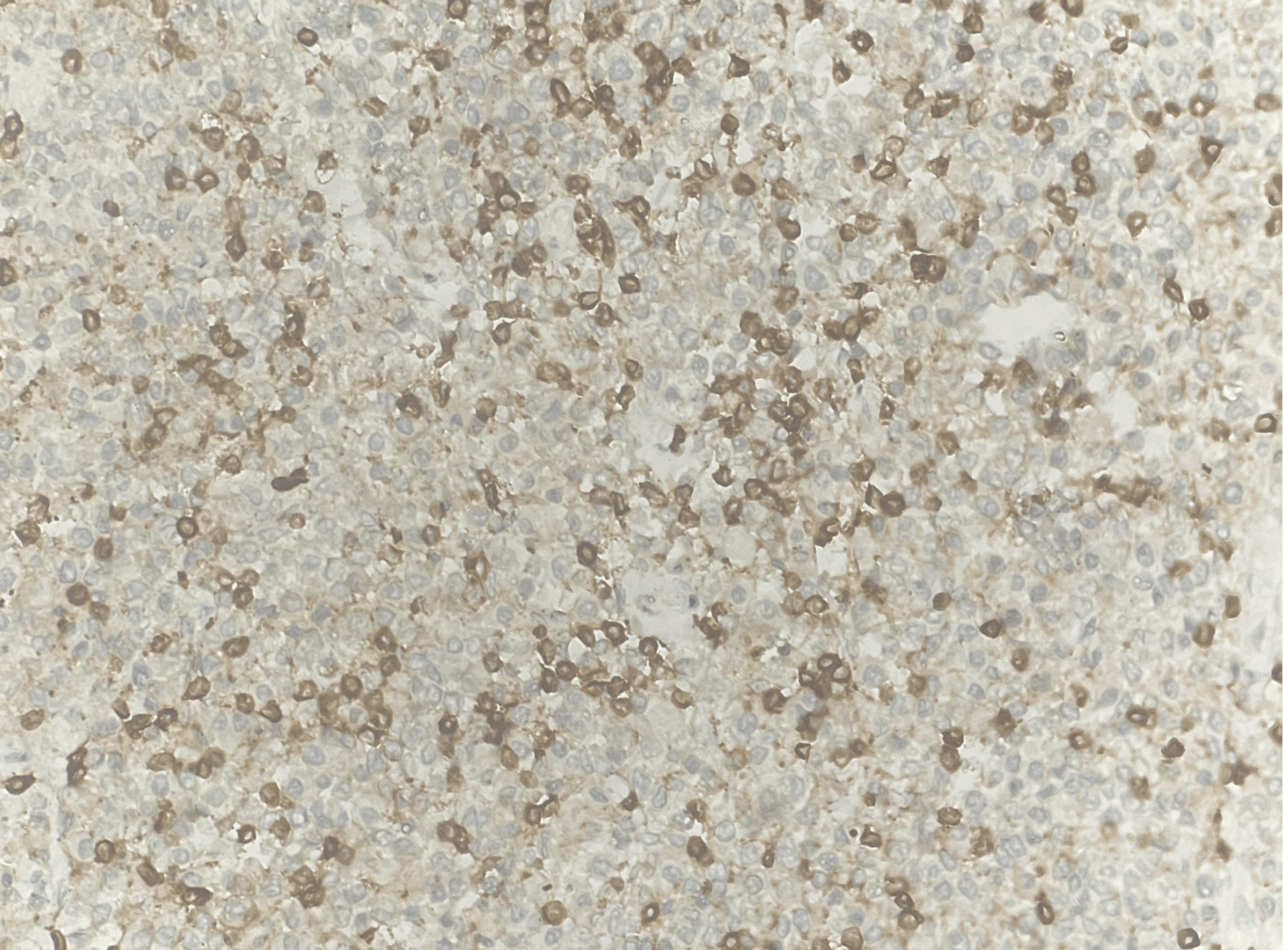
Tumor in the appendix - CD3 immunohistochemistry (10x magnification) CD3 showed positivity in scattered reactive lymphoid cells.

CD3 showed positivity in scattered reactive lymphoid cells (Figure 6).

**Figure 7:**
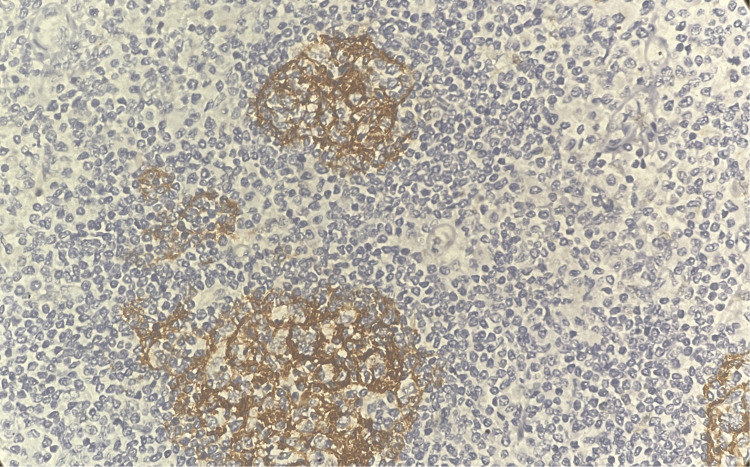
Tumor in the appendix - CD23 immunohistochemistry (10x magnification) CD23 was negative in the lymphoid cells but positive in the follicular dendritic cell meshwork of the residual follicle.

Further, IHC was done for cyclin D1, CD5, CD23, CD10, Bcl6, and Bcl2. Bcl2 and cyclin D1 were not expressed in the lymphoid cells. CD10 and Bcl6 were negative. CD23 was negative in the lymphoid cells but was found to be positive in the follicular dendritic cell meshwork of the residual follicle (Figure 7). 

**Figure 8:**
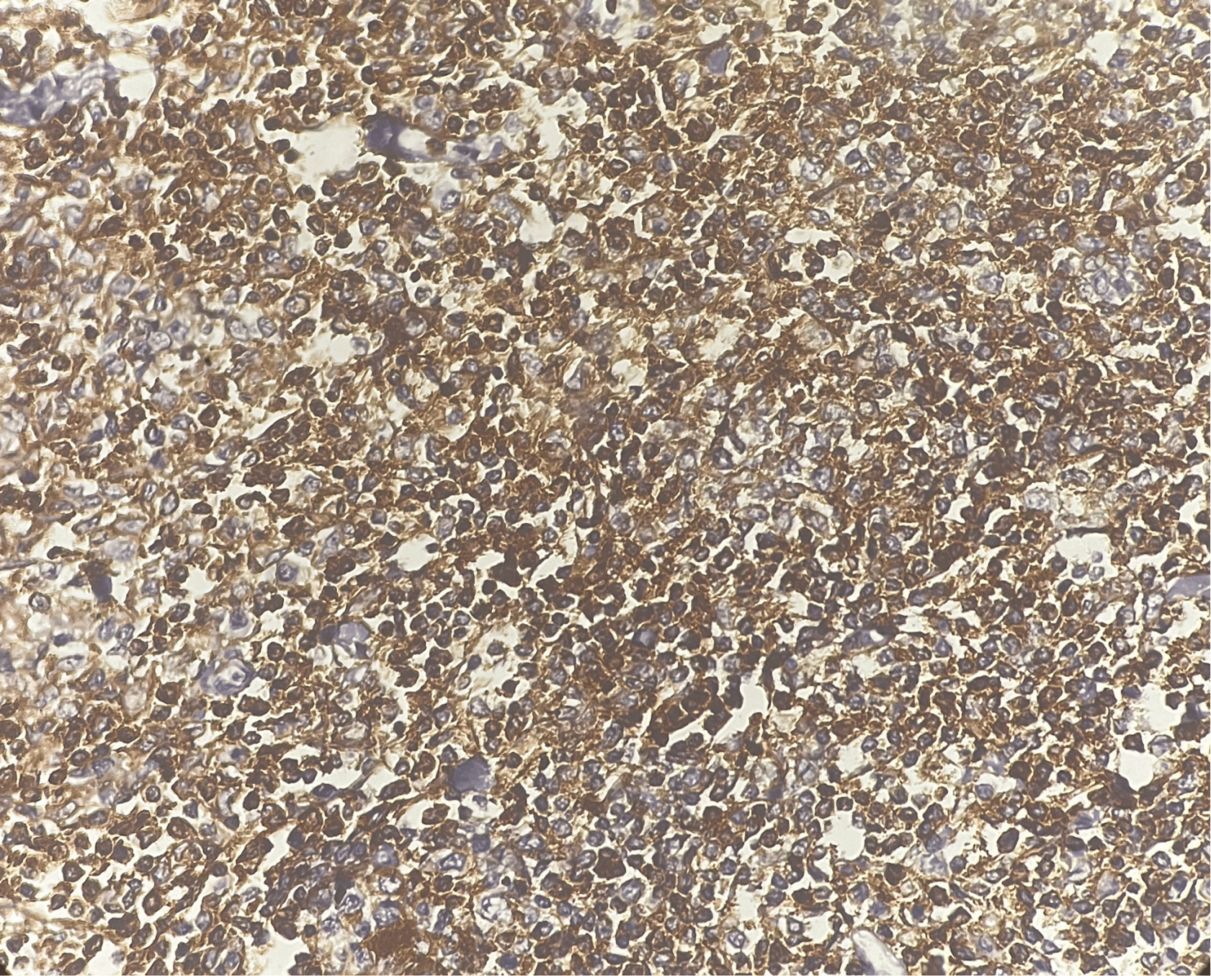
Tumor in the appendix - CD5 immunohistochemistry (10x magnification) CD5 showed strong positivity in 80% of lymphoid cells.

CD5 showed strong positivity in 80% of lymphoid cells (Figure 8). 

**Figure 9:**
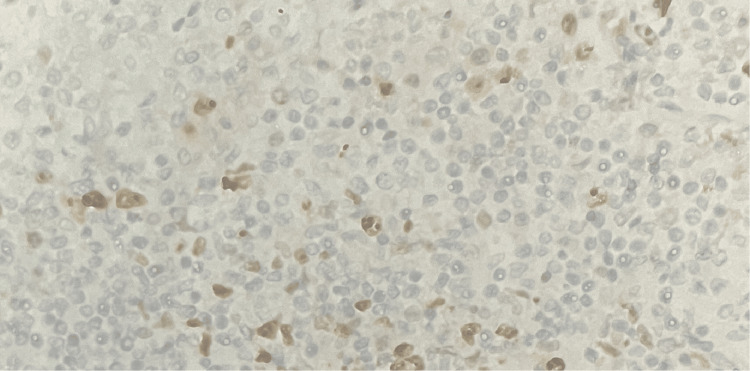
Tumor in the appendix - Ki67 immunohistochemistry (10x magnification) The proliferation index with Ki67 was found to be 10%.

The proliferation index with Ki67 was found to be 10% (Figure 9). 

**Figure 10:**
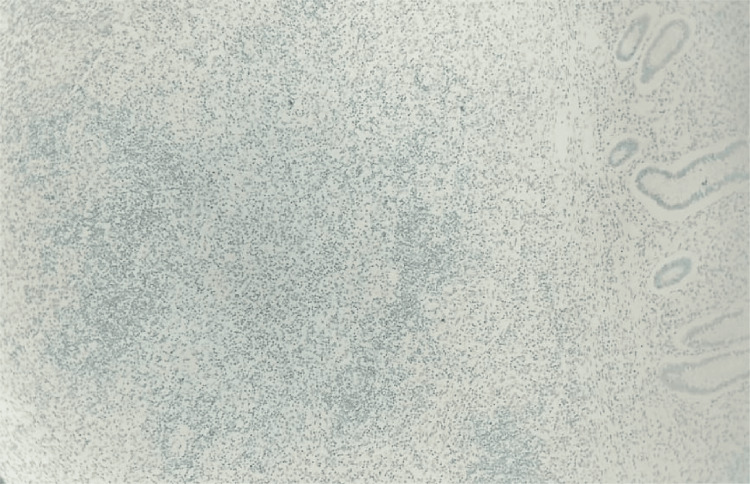
Tumor in the appendix - LEF1 immunohistochemistry (10x magnification) LEF1 was negative in the tumor cells.

Further, IHC for LEF1 was done on the tumor in the appendix, and it was found to be negative, thus, ruling out small lymphocytic lymphoma (SLL) (Figure 10). 

**Figure 11:**
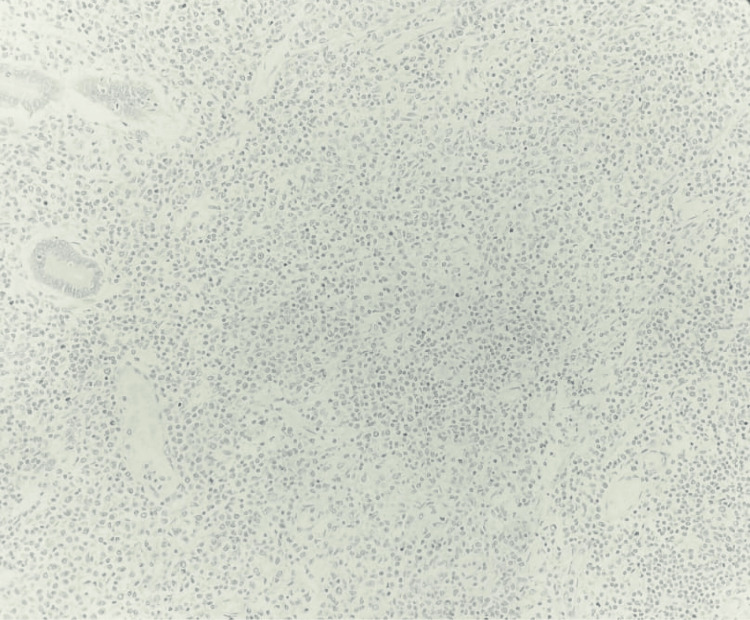
Tumor in the appendix - SOX11 immunohistochemistry (10x magnification) SOX11 was negative in the tumor cells.

Additionally, IHC for SOX11 was done on the tumor in the appendix. It was also found to be negative, ruling out mantle cell lymphoma as well (Figure 11). 

These features confirmed CD5-positive extranodal marginal zone lymphoma of the MALT involving the appendix and the adjacent caecal wall.

Considering CD20 and Ki67, an initial diagnosis of low-grade NHL B-cell origin was considered. In view of CD5 positivity in low-grade lymphoma, three differential diagnoses including SLL, mantle zone lymphoma, and CD5-positive extranodal marginal zone lymphoma were considered. CD23 negativity and LEF1 negativity ruled out the possibility of SLL. Cyclin D1 and Bcl2 negativity, along with SOX11 negativity, excluded the diagnosis of mantle zone lymphoma. Correlating IHC with histomorphology, the final diagnosis of CD5-positive extranodal marginal zone lymphoma was made. The patient was given two cycles of rituximab plus cyclophosphamide, doxorubicin, vincristine, and prednisone (R-CHOP) regimen chemotherapy.

One month postoperatively, the patient presented with complaints of pain in the right leg for one week associated with discoloration of the right feet and toes. On examination, the right foot was dusky and cold to touch and the patient had a foot drop. In her right leg, the common femoral, popliteal, anterior and posterior tibial, and dorsalis pedis artery pulses were not palpable. Prothrombin time and international normalized ratio (PT/INR) was 11.4 seconds, INR was 0.99, activated partial thromboplastin time (APTT) was 20.9 seconds, and D-dimer was 1894 ng/ml. CT angiogram showed thrombo-occlusive disease in the right lower limb with soft calcific plaques involving the right common iliac, internal iliac, proximal external iliac, and common femoral and superficial femoral arteries, causing complete luminal narrowing. However, a mid-distal passage of contrast was seen. The patient was taken up for a right lower limb thromboembolectomy, and the thrombi were removed. The patient was given heparin infusion along with other oral antiplatelet medications and statins. Post-surgery, the patient developed right foot gangrene and hence a below-knee amputation was done for the patient. However, a few weeks post-surgery, the patient succumbed to the illness.

## Discussion

Malignancies of the appendix are seen in less than 1% of the appendicectomy specimens [7]. Based on the data from the Surveillance, Epidemiology, and End Results (SEER) database of appendiceal malignancies from 1973 to 2004, adenocarcinoma of the appendix accounted for 65.4% of appendiceal cancers, with neuroendocrine neoplasms of the appendix coming in second. The five-year survival rates were found to be 1.7% for lymphoma, 20.3% for signet ring cell carcinoma, 59.5% for mucinous cystadenocarcinoma, and 47.7% for mucinous adenocarcinoma [7,9].

NHL is a common neoplastic disease involving the lymphoid tissue. Extranodal lymphoma is the term used for NHL that originates outside the lymph nodes [4]. The stomach is the most often encountered extranodal location in NHL cases. The small intestine, pharynx, colon, and esophagus are the next most frequently encountered sites [4,7]. Primary appendiceal lymphoma is quite uncommon. In general, primary appendiceal NHL accounts for 1.3% to 2.6% of all GIT lymphomas [9]. Primary lymphomas of the appendix are almost exclusively NHL B-cell type lymphoma [10] as seen in our case. The male and female appendiceal lymphoma ratio is 1.5:1, indicating a higher incidence of this disease in the male population. With 18 years being the mean age of occurrence for appendix lymphomas, it is more common in young individuals [7,11]. However, our patient was an elderly 78-year-old female.

IHC is helpful in distinguishing B-lymphocytes from T-lymphocytes [10], as in our case, where IHC showed features conclusive of NHL B-cell origin. Further, IHC was done for cyclin D1, CD5, CD23, CD10, Bcl6, Bcl2, LEF1, and SOX11 on the tumor in the appendix. The features were conclusive of CD5-positive extranodal marginal zone lymphoma of the MALT.

Treatment for most tumors in the appendix is surgery. However, lymphoma requires cyclophosphamide, doxorubicin, vincristine, and prednisone (CHOP) regimen or R-CHOP regimen chemotherapy [12,13]. In our patient, caecal involvement was present, hence right hemicolectomy was done, following which the patient was given two cycles of R-CHOP regimen chemotherapy.

When arterial thrombosis coexists with malignancy, the prognosis is poor. The outcome of surgery for the same is poor as well. Thus, arterial thrombosis in the setting of malignancy has an overall bad prognosis [14]. Thrombo-occlusive disease in this patient could have occurred as a post-surgical complication due to atherosclerosis, which is the most common cause. However, thrombophilia in a setting of lymphoma, although a rare association, cannot be ruled out in this patient.

## Conclusions

This report emphasizes how challenging it might be to diagnose appendicular lymphoma preoperatively. We further stress that lymphoma should be taken into consideration as a differential diagnosis in older individuals who come with an appendiceal mass. Furthermore, the prognosis is not good when cancer and arterial thrombosis coexist. Additionally, the results of surgery for the same are also not good, as seen in the present case. Thus, the overall prognosis for arterial thrombosis in the presence of malignancy is often poor.

Primary appendiceal lymphoma can closely mimic symptoms of acute appendicitis. While differentiating them through CT scans alone can be challenging, some telltale signs can point toward lymphoma. Regardless of whether the disease is localized or widespread, early diagnosis offers the best chance for a positive outcome.
